# Protective and Detoxifying Effects of Resveratrol on Zearalenone-Mediated Toxicity: A Review

**DOI:** 10.3390/ijms252011003

**Published:** 2024-10-13

**Authors:** Qiongxia Lv, Wenjing Xu, Fan Yang, Jiahui Li, Wenjuan Wei, Xiaoguang Chen, Yumei Liu, Ziqiang Zhang

**Affiliations:** College of Animal Science and Technology, Henan University of Science and Technology, No.263, Kaiyuan Avenue, Luoyang 471023, China; xwenjing0223@163.com (W.X.);

**Keywords:** resveratrol, zearalenone, toxicity, molecular mechanism

## Abstract

Zearalenone (ZEA) is a mycotoxin produced by Fusarium spp. fungi and is widely found in moldy corn, wheat, barley, and other grains. ZEA is distributed to the whole body via blood circulation after metabolic transformation in animals. Through oxidative stress, immunosuppression, apoptosis, autophagy, and mitochondrial dysfunction, ZEA leads to hepatitis, neurodegenerative diseases, cancer, abortion, and stillbirth in female animals, and decreased sperm motility in male animals. In recent years, due to the influence of climate, storage facilities, and other factors, the problem of ZEA pollution in global food crops has become particularly prominent, resulting in serious problems for the animal husbandry and feed industries, and threatening human health. Resveratrol (RSV) is a natural product with therapeutic activities such as anti-inflammatory, antioxidant, and anticancer properties. RSV can alleviate ZEA-induced toxic effects by targeting signaling pathways such as NF-κB, Nrf2/Keap1, and PI3K/AKT/mTOR via attenuating oxidative damage, inflammatory response, and apoptosis, and regulating cellular autophagy. Therefore, this paper provides a review of the protective effect of RSV against ZEA-induced toxicity and its molecular mechanism, and discusses the safety and potential clinical applications of RSV in the search for natural mycotoxin detoxification agents.

## 1. Introduction

Mycotoxins are toxic metabolites produced by molds, and more than 300 mycotoxins have been identified. About a quarter of the agricultural products in the world are contaminated with mycotoxins to varying degrees each year [1]. Mycotoxins contaminating animal feed ingredients are mainly from the genera *Penicillium*, *Aspergillus*, and *Fusarium* [2]. The main mycotoxins produced by these molds are aflatoxins (AFs), deoxynivalenol (DON), and zearalenone (ZEA) [3]. Rodrigues et al. showed a 25% positive detection rate for ZEA and a 19% positive detection rate for AFs in a survey of mycotoxin levels in commercial feeds and feed ingredients from the Middle East and Africa [4]. Liu et al. investigated mycotoxin in feed ingredients and full-price feeds from central China; the positive detection rate of ZEA was 100%, with a maximum positive level of 651.8 μg/kg [5]. Mycotoxicosis can lead to a range of toxic effects, including ZEA-induced precocious puberty, reduced embryo survival, reduced sperm count in males, and toxic effects on organs such as the liver and intestines [6]. Globally, AFs have been associated with 28.20% of hepatocellular carcinoma (HCC) cases [7], and individuals consuming DON-contaminated foods may experience vomiting syndrome [8].

**Figure 1:**
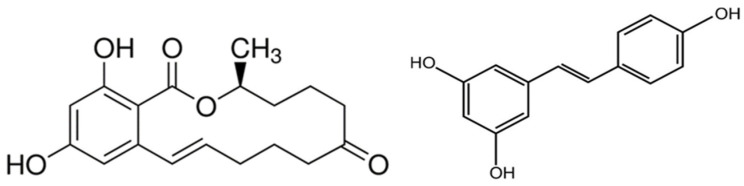
Chemical structure of ZEA and RSV [35,36].

Crops contaminated with *Fusarium* and *Gibberellins* due to improper storage or climatic reasons may be used as feed ingredients [9]. ZEA, known as F-2 toxin, is a non-steroidal estrogenic mycotoxin (Figure 1) [10]. ZEA is classified as a Group 3 carcinogen by IARC, and the European Commission has fixed the maximum level of ZEA at 20–100 ppb depending on the food involved (ECNo.1126/2007) [11]. ZEA derivatives include α/β-zearalenol (a/β-ZOL) and α/β-zearalanol (a/β-ZAL). Thus, ZEA can competitively bind to the 17-β estrogen receptor in humans and animals, triggering a range of toxic effects [12]. Studies have shown that the affinity of ZEA and its derivatives for the β-estradiol receptor is ranked α-ZAL > α-ZOL > β-ZAL > ZEA> β-ZOL [13]. The greater the affinity for the estrogen receptor, the greater the estrogenic activity and the greater the toxicity. It was shown that ZEA affects meiotic maturation of porcine oocytes through its effects on protein synthesis, transport, and degradation [14]. It is worth noting that in other countries (such as Japan), ZEA intake from diets may affect ovarian sinus follicle counts in postpartum heifers even if it is below the permissible ZEA contamination threshold (<1 ppm), but it does not affect their fertility [15]. Exploring the effect of gut microbiota on ZEA by inhibiting gut microbes in broiler chickens showed that microbial inhibition resulted in a significant increase in the levels of ZEA and its metabolites, as well as an increase in liver injury [16]. Due to the high risk levels and toxicity of ZEA, scientists from around the world have been investigating the molecular pathways and detoxification methods associated with ZEA toxicity [17,18].

In order to avoid contamination by ZEA and to achieve complete detoxification, it is necessary to understand the synthesis, toxicity, and molecular mechanisms of ZEA. Commonly used detoxification methods include biological, physical, and chemical methods, but due to high side effects, incomplete detoxification, and the high price, scientists are working on exploring more suitable methods to control mycotoxins [19,20]. Natural products have the advantages of good biodegradability, efficient detoxification, no toxic side effects, and a widespread presence in nature, and therefore have good prospects for application in mycotoxin detoxification [21]. Several natural products or active compounds, including vitamins, baicalin, saffron, and pro-anthocyanidins, have been found to attenuate ZEA-induced organ damage [22,23,24,25]. Although some natural products have been found to mitigate the toxic effects of ZEA, resveratrol (RSV) is one of the typical representatives due to its low cost and high safety. RSV is a non-flavonoid polyphenolic compound with a molecular formula of C_14_H_12_O_3_ (Figure 1). The pure RSV is colorless and odorless crystals, easily soluble in organic solvents such as ethanol, methanol and ether, with a melting point of 256–258 °C [26]. RSV has antioxidant, anti-inflammatory, anticancer, and cardiovascular protective effects. RSV is present in a wide range of plants, such as berries, grapes, and peanuts. In grape skins it is present in the range of 50–100 μg/g, and in red wines in the range of 1.5–12 mg/L [27]. It is evident that RSV is widely available and possesses three phenolic hydroxyl groups, which offer advantages such as an anti-inflammatory and antioxidant protective agent. RSV has antimicrobial activity against *Fusarium* and achieves its antimicrobial effect by disrupting the structure of fungal cell walls and cell membranes, as well as interfering with fungal metabolic processes, thus inhibiting the production of ZEA in feed and food [28]. Animal in vivo and in vitro experimental studies have reported that RSV supplementation can effectively attenuate ZEA-induced renal dysfunction, intestinal damage, and reproductive system damage by inhibiting oxidative stress (OS), apoptosis, and inflammatory responses [29,30,31]. Importantly, RSV has been shown to be safe and reliable in human clinical trials or animal testing [32]. Only high oral doses of RSV (2000 mg twice daily) have been reported to cause minor to medium gastrointestinal complaints in healthy individuals [33]. Similarly, no negative side effects were observed when RSV was administered orally to laboratory animals for 90 days at a dose of 200 mg/kg/day in rats and 600 mg/kg/day in dogs [34]. Therefore, the aim of this review is to provide a complete outline of the protection of RSV against ZEA poisoning, including its molecular mechanisms and clinical significance, with a view to informing future research and intervention aimed at reducing the health risks of ZEA.

## 2. ZEA Toxic Effects and Its Molecular Mechanism Research Progress

**Figure 2:**
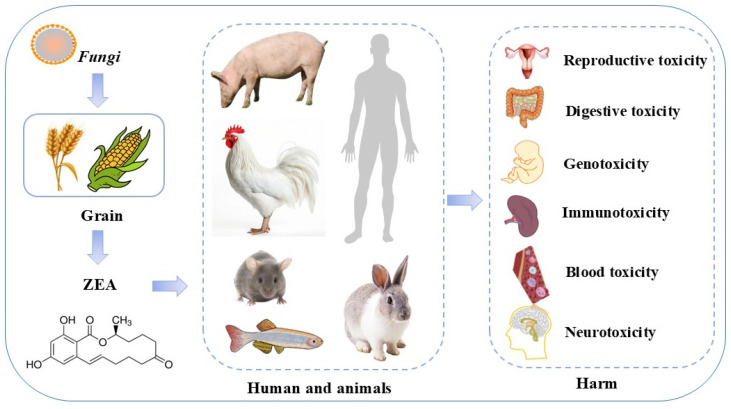
Toxic effects of ZEA.

ZEA and its metabolites also exhibit many toxic side effects such as liver, kidney, spleen, brain, intestinal, testicular, and ovarian tissue toxicity, as well as mutagenicity, teratogenicity, and carcinogenicity (Figure 2) [37,38,39,40,41,42,43]. The main target organ of ZEA action is the reproductive system of females. The structure of ZEA is similar to that of endogenous estrogens, and it enters the body and competitively binds to estrogen receptors, thus triggering a series of estrogen-like effects, mainly disrupting the secretion of reproductive hormones in animals, inducing OS and apoptosis in ovarian granulosa cells, causing damage to organs such as the liver [44] and triggering tumors, among others [45]. Thus, ZEA exposure is associated with tumorigenesis in humans and other animals. Excessive reactive oxygen species (ROS) production, DNA damage, OS, apoptosis, cellular autophagy, mitochondrial dysfunction, and inflammatory responses have all been linked to ZEA-induced cytotoxicity or cell death pathways [46,47,48,49]. Further explorations by scientists have revealed many signaling pathways involved in ZEA-induced toxic effects in in vivo and in vitro models, such as nuclear factor erythroid 2 related factor 2 (Nrf2)/Kelch-like ECH-Associating protein 1 (Keap1), Phosphoinositide 3-kinase (PI3K)/protein kinase B (Akt), Adenosine monophosphate (AMP)-activated protein kinase (AMPK)/mechanistic target of rapamycin kinase (mTOR), Ras/Raf/extracellular-signal-regulated kinase (ERK), Mitogen-activated protein kinases (MAPK), Nuclear factor kappa-light-chain-enhancer of activated B cells (Nf-κB), and Wnt-1/β-catenin signaling pathway [50,51,52,53,54,55,56]. Thus, by targeting these keys signaling pathways, it may provide a key target for preventing or treating the deleterious consequences caused by ZEA in humans and animals.

## 3. Biological Properties of RSV

**Figure 3:**
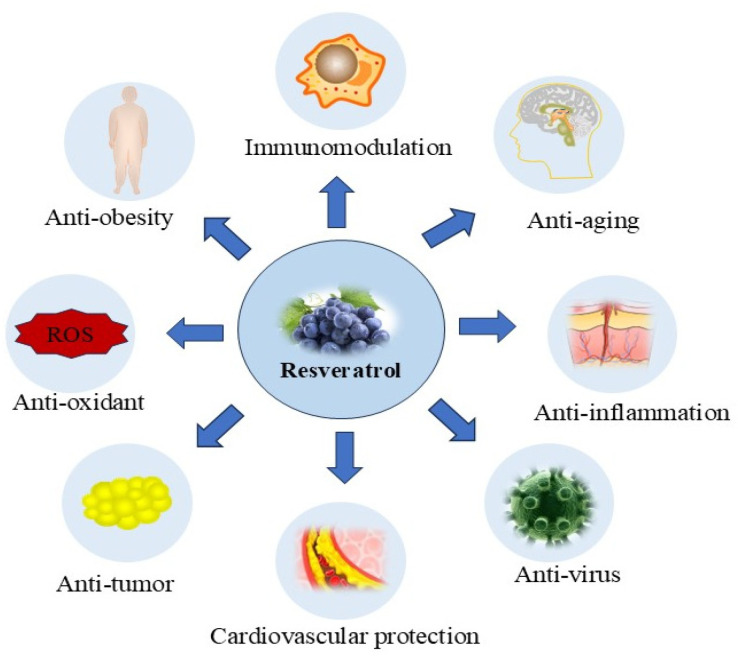
Schematic representation of the biological effects of RSV.

RSV is a phenolic substance originally isolated from quinoa, and can be extracted from more than 70 plants and mainly exists in grapes [57]. RSV is also found in products such as red wine, pistachios, cranberries, pomegranates, chocolate, and cocoa powder [58]. Adequate RSV can also be obtained through nutraceuticals and supplements. RSV usually exists in the form of cis-and trans-isomers, and its trans-isomers have higher stability and biological activity. Therefore, the application of trans-isomers for treatment has become the focus of research [59]. RSV has a variety of biological functions such as anti-inflammatory, antioxidant, anti-tumor, anti-obesity, enhancement of immunity, and prevention of neurodegenerative diseases and cardiovascular disease (Figure 3) [60]. RSV also plays an important role in regulating lipid peroxidation, protein carbonylation, and mitochondrial permeability, ultimately preventing apoptosis, iron death, cell necrosis, etc. [61]. It was found that RSV works by targeting a variety of signaling pathways, including Wnt/β-catenin (Wingless/β-catenin), PI3K/AKT/mTOR, Nrf2/Keap1, MAPK, NF-κB, tumor necrosis factor β (TNF-β), Signal Transducer And Activator Of Transcription 3 (STAT3), Transforming growth factor-β1 (TGF-β1) /Smads, and Silent information regulator 1 (SIRT1)/Forkhead box O3 (FOXO3A) [62,63,64,65,66,67,68,69], ultimately inhibiting hepatotoxicity, nephrotoxicity, and hematotoxicity caused by mycotoxins (e.g., AFs, ochratoxin A, DON), heavy metals (e.g., cadmium, arsenic, mercury), and pathogenic agents (e.g., *Staphylococcus aureus*, pathogenic *Escherichia coli*, *Klebsiella pneumoniae*), among others [70,71,72,73,74,75,76,77,78]. In addition, multiple studies have demonstrated the high therapeutic value of RSV in the treatment of a number of unique chronic diseases, including cancer, nervous system disorders, cardiovascular diseases, and metabolic disorders [79]. Several in vitro and in vivo animal studies have also confirmed that RSV provides potent protection against ZEA-induced hepatic injury, mutagenicity, renal dysfunction, intestinal damage, and reproductive toxicity by modulating several of the above targets [30,80,81,82]. The detailed mechanisms by which RSV exerts its biological effects are discussed below.

## 4. Protective Effects of RSV against ZEA Toxicity and Its Molecular Mechanism

Table 1 summarizes in vitro and in vivo studies involving the protective effects of RSV against ZEA-induced toxicity. The molecular mechanisms are mainly involved in the inhibition of OS, inflammatory response, apoptosis, attenuation of mitochondrial dysfunction, modulation of immune response, and alleviation of DNA damage. Subsequent sections will delve into the exact molecular mechanisms of the protective effects of RSV.

### 4.1. RSV Ameliorates ZEA-Induced Oxidative Stress

Produce of excess ROS be cells leads to an imbalance between the oxidative and antioxidant systems in the body, which in turn triggers OS [84]. OS is among the key molecular mechanisms for the undesirable effects of many toxins or chemically harmful substances (e.g., heavy metals, mycotoxins, antibiotics, and pesticides) [85]. ROS include a variety of free radicals such as hydroxyl radicals, hydrogen peroxide, and nitric oxide [86]. In usual circumstances, endogenous cellular antioxidants or antioxidant enzymes, such as SOD, GPX, CAT, and GSH, effectively counteract these free radicals [87]. The study showed that ZEA and its derivatives can initiate OS by leading to the generation of ROS free radicals or by decreasing the activity of intracellular antioxidants or antioxidant enzymes [88]. ROS production by ZEA is partly attributed to its metabolic processes in tissues. Increased ROS in cells can cause damage to intracellular lipids, proteins, and DNA, resulting in a variety of death modes such as apoptosis, autophagy, iron death, and cell necrosis [89].

Studies have found that daily oral gavage of 40 mg/kg BW ZEA to male BALB/c mice for 12 days caused significant intestinal oxidative damage, as evidenced by a significant decrease in antioxidant enzyme activities (e.g., T-SOD, CAT) and elevated MDA content in intestinal tissues. Meanwhile, oral gavage RSV supplementation significantly inhibited ZEA-induced OS by upregulating the activity of antioxidant enzymes (e.g., T-SOD, CAT) and decreasing the content of MDA [30]. In addition, RSV supplementation significantly ameliorated ZEA-induced cytotoxicity in human embryonic renal cells by inhibiting ROS and OS production through upregulation of antioxidant enzyme activity (MnSOD) or free radical scavenging levels [29]. In conclusion, RSV activates the antioxidant system of the body and plays an important role in protecting the body from ZEA-induced toxicity.

Nrf2 is a key transcription factor in the cellular modulation of OS [90]. Nrf2 activation produces over 200 genes engaged in antioxidant, anti-inflammatory, and xenobiotic metabolic processes, which protect cells from foreign toxins [91]. In the normal physiological microenvironment, Nrf2 is located in the cytoplasm and interacts with Keap1, which is ubiquitinated and degraded via the ubiquitin–proteasome system [92]. In OS conditions, ROS and electrophiles bind directly to Keap1 at multiple sites, enabling Nrf2 to detach from Keap1, translocate to the nucleus, and subsequently induce the expression of protective genes in response to various stresses [93]. Previous studies have shown that exposure to ZEA significantly inhibits *Nrf2* gene expression, leading to increased sensitivity to ZEA in Nrf2 knockout mice and emphasizing the critical role of Nrf2 as a ZEA target [94]. Exposure to ZEA suppressed the expression of Nrf2 and its downstream proteins, such as heme oxygenase-1 (HO-1), quinone oxidoreductase 1 (NQO1), SOD, and CAT, leading to OS in rabbit liver, porcine intestine, and rat liver and kidney tissues [95,96,97]. RSV has been shown to be an activator of Nrf2 [98]. RSV binds directly to Keap1, thereby reducing the tightness of the Keap1-Nrf2 protein–protein interaction [99]. Supplementation with RSV can greatly improve the expression of Nrf2, which effectively activates the Nrf2/Keap1 signaling pathway, leading to an increase in the expression of its downstream proteins [100]. These findings indicate that the activation of the Nrf2/Keap1 signaling pathway is crucial for the protective action of RSV against OS caused by ZEA exposure. Previous research has demonstrated that the activation of the Nrf2/Keap1 pathway induced by RSV may be modulated by p62 and SIRT1 [101,102]. Nevertheless, the mechanism of direct interaction between RSV and Keap1 remains unclear, and further studies are needed to elucidate the precise molecular mechanism by which RSV activates the Nrf2/Keap1 pathway.

**Figure 4:**
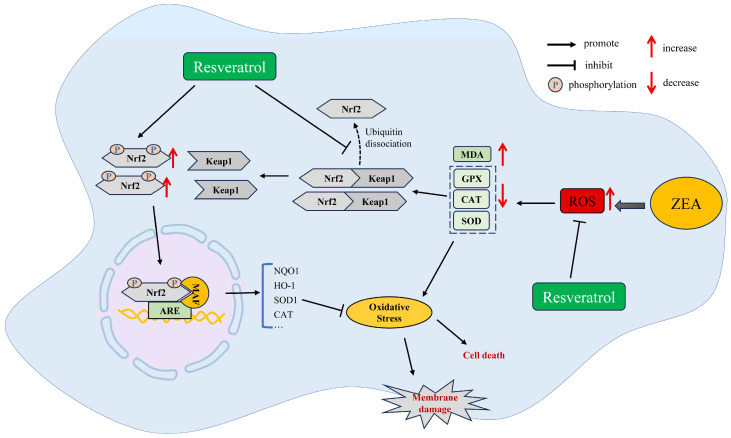
RSV ameliorates ZEA-induced toxicity by inhibiting ROS and OS production and activating Nrf2-mediated antioxidant defenses.

In conclusion, as shown in Figure 4, RSV supplementation protects various tissues from ZEA-induced OS through direct scavenging of free radicals, enhancement of antioxidant enzyme activity, and activation of the Nrf2/Keap1 pathway.

### 4.2. RSV Ameliorates Immunosuppression and Inflammatory Response Induced by ZEA Exposure

ZEA plays a significant role in the immune response, which constitutes the primary defense system of all mammals against pathogens, toxins, and various other antigenic substances [103]. Long-term exposure to ZEA can lead to immunosuppression, manifested by the inhibition of immune cells, immune factors (such as IL-10 and TNF-α, etc.), and production of lymphocytes (B cells and T cells), resulting in reduced spleen weight, decreased number of CD4+ cells in tissues, and inhibition of immune cell proliferation, and possibly leading to the occurrence of various inflammation-related diseases (such as neurodegenerative diseases, chronic enteritis, and liver cancer) [104,105], making host cells more susceptible to viral infection. Previous studies have shown that the inflammatory response and immunotoxicity induced by ZEA may involve multiple pathways, such as NF-kB, Toll-like receptor 4 (TLR4), NRLP3, and MAPK pathways. Detailed molecular mechanisms are outlined in the following text.

NF-κB plays a crucial role in the cellular inflammatory response, immune response, and other processes. There are many activators of the NF-κB pathway, such as TNF-α, IL-1β, IL-6, IL-8, IL-12, and chemokines [106]. After feeding weaned piglets with feed contaminated with ZEA for 18 days, the expression levels of pro-inflammatory factors TNF-α, IL-8, IL-6, and IL-1β significantly increased, and the MAPK signaling pathway was activated, leading to a notable inflammatory response in the spleen [107]. Previous research has shown that ZEA can inhibit the activation of T cells and the expression of activation signals CD69, CD25, and CD71 at various stages, and can also inhibit the expression of key proteins involved in T cell activation such as LAT, Lck, and Zap-70 [108], indicating that ZEA induces immunotoxicity. These findings further confirm that the activation of the NF-kB signaling pathway may play a key role in the inflammatory response induced by ZEA. Previous studies have found that daily intragastric administration of 4.5 mg/kg BW of ZEA to mice for 7 days resulted in significant increases in the levels of IL-1β and IL-18. The protein expression levels of NLRP3, ASC, and Caspase1 also significantly increased [109]. ZEA exerts direct toxic effects on the intestinal epithelial barrier and promotes the accumulation of ROS. Macrophages are stimulated by ROS, thereby promoting the transcription of pro-IL-1β and pro-IL-18. ZEA activates Caspase-1 through the NLRP3 inflammasome complex, cleaving pro-IL-1β and pro-IL-18 into their biologically active forms, thereby initiating a cascade of intestinal inflammatory responses. TLR4 is an important member of the TLR family expressed in many cells, including renal, heart, adipose tissue, and intestinal epithelial cells, and mediating inflammatory processes by recognizing endogenous and exogenous ligands [110]. After being stimulated by invading pathogens, TLR4 is activated, leading to signal transduction into the cell. The activated TLR4 binds to the adapter protein MyD88, which in turn activates NF-kB p65 through a cascade of signaling reactions [111]. Via its nuclear localization sequence (NLS), NF-kB p65 translocates from the cytoplasm to the nucleus, where it initiates the transcription, synthesis, and release of mRNA for IL-1, IL-6, IL-8, IL-10, TNF-a, and other inflammatory factors to stimulate local or systemic inflammatory responses [112]. When pregnant rats were fed diets containing 0.3, 48.5, 97.6, or 146 mg/kg of ZEA, respectively, ZEA increased the protein expression of IL-1β, TNF-α, TLR4, and NF-kB p65 in a dose-dependent manner [113]. ZEA can induce the upregulation of *TLR4* gene expression level, thereby activating its signaling cascade, and promoting the release of inflammatory factors.

**Figure 5:**
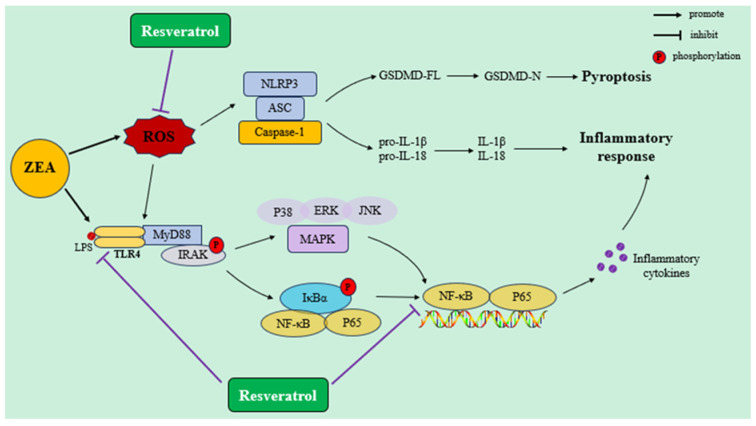
RSV alleviates ZEA-induced inflammatory response by regulating NLRP3, TLR4, and NF-κB signaling pathways.

Multiple studies have demonstrated that RSV has immune-regulating and anti-inflammatory properties, which can regulate immunity by interfering with immune cell regulation, promoting the synthesis and gene expression of pro-inflammatory cytokines, and directly or indirectly targeting various signaling molecules (such as NF-kB, IL-6, and IL-8) and immune cells [114]. Supplementation with different concentrations of RSV significantly reduced the elevation of pro-inflammatory factors TNF-α, IL-6, and IL-1β in the serum of mice induced by 40 mg/kg BW ZEA, and decreased inflammation occurrence by blocking the translocation of NF-κBp65 [30]. RSV is also an inhibitor of the transcription factor NF-κB, which suppresses the expression of various inflammatory factors, including TNF-α and IL-1β [115]. Inhibition of the NF-κB pathway by RSV can be achieved by blocking the TLR 4 and P38 MAPK pathways [116,117]. Furthermore, excessive generation of ROS can exacerbate the production of pro-inflammatory cytokines and chemokines by triggering the NLRP3, TLR4, NF-κB, and MAPK pathways [118,119]. In summary, these results indicate that RSV can alleviate ZEA-induced inflammatory response by inhibiting the TLR4, NF-κB, and MAPK pathways and reducing ROS generation. However, the exact molecular mechanisms by which RSV effectively combats the ZEA-induced inflammatory response are still not fully understood and require further comprehensive investigation (Figure 5).

### 4.3. RSV Improves Mitochondrial Dysfunction and Apoptosis Induced by ZEA

Mitochondria can promote the production of adenosine triphosphate (ATP) through oxidative phosphorylation (OXPHOS), playing a crucial role in maintaining cellular homeostasis, as well as participating in the regulation of apoptosis and autophagy processes [120]. Abnormal mitochondrial membrane potential and electron transfer can cause mitochondrial dysfunction, which is associated with excessive ROS production. Excessive ROS production can lead to damage to lipids, nucleic acids, and proteins, and ultimately result in cell apoptosis and necrosis, triggering neurodegeneration [121]. Studies have shown that ZEA can induce mitochondrial dysfunction, manifested as disruption of mitochondrial membrane potential and the electron transport chain, and reduced activity of mitochondrial respiratory chain complexes (COX I-IV), leading to cellular toxicity and tissue damage [122,123,124]. Research has indicated that exposure to ZEA can damage mitochondrial ultrastructure, reduce the expression levels of related proteins on the mitochondrial membrane (Mfn2, VDAC1), and inhibit the synthesis of mitochondrial biofilms, leading to mitochondrial dysfunction [122]. ZEA can also induce mitochondrial dysfunction in goat endometrial stromal cells by reducing mitochondrial membrane potential, inhibiting mitochondrial biogenesis and OXPHOS-mediated ATP generation [125]. Furthermore, exposure to ZEA can also decrease the expression of proteins related to the tricarboxylic acid (TCA) cycle and oxidative phosphorylation, affecting mitochondrial metabolic function [126]. Another study has found that exposure to ZEA significantly reduces the expression levels of DRP1 and MFN1 proteins in porcine fetal support cells, causing mitochondrial enlargement and ultimately leading to mitochondrial biosynthesis disorders [127]. In summary, the mechanisms by which ZEA induces mitochondrial dysfunction may include damage to mitochondrial ultrastructure, decreased mitochondrial membrane potential, inhibition of the TCA cycle and oxidative phosphorylation, disruption of mitochondrial biosynthesis, and reduced ATP production.

Apoptosis represents a type of programmed cell death mechanism that can be initiated by diverse external factors, encompassing fungal toxins, heavy metal exposure, ultraviolet radiation, and pathogenic microorganisms [128]. Previous studies have found that mitochondrial dysfunction in mammalian cells can lead to the activation of the mitochondrial apoptotic pathway, resulting in Caspase-dependent apoptosis [129]. The initiation of the mitochondrial apoptotic pathway usually depends on the proteins of the Bcl-2 family, primarily through the expression of pro-apoptotic proteins (such as Bax, Bak) and anti-apoptotic proteins (Bcl-2, Bcl-xL) [130]. Cell death induced by apoptosis is typically triggered by mitochondrial outer membrane permeabilization (MOMP), where pro-apoptotic factors are released from mitochondria in a cascading manner into the intermembrane space, activating Caspase-9, Caspase-3, etc., leading to the opening of the mitochondrial permeability transition pore, resulting in a reduction in mitochondrial membrane potential and ultimately causing cell apoptosis [131]. Research has found that treatment with ZEA can increase the protein expression levels of Bax, decrease the protein expression levels of Bcl-2, increase the activation of Caspase-9, and promote the release of Cyt C from mitochondria to the cytoplasm, triggering cell apoptosis [132]. In addition, ZEA-induced cell apoptosis also involves other signaling pathways, including the Fas-Fas ligand, ATP/AMPK, RIPK1, and ERK1/2/p53/Caspase 3. These pathways play important roles in regulating the mitochondrial apoptosis pathway [133,134,135].

It has been reported that supplementing RSV can inhibit the reduction in mitochondrial membrane potential, reduce mitochondrial ROS production, control the number of mitochondria, alleviate damage to mitochondria from external stimuli, and enhance mitochondrial biogenesis by acting on its major effectors, including SIRT1, AMPK, Nrf1, and Nrf2 [136]. Supplementing RSV can reduce the Bax/Bcl-2 ratio, decrease MOMP, reduce the release of Cyt C, and ultimately improve cell apoptosis caused by exogenous or endogenous stimuli. Research has found that RSV can decrease the expression of Caspase-3, Caspase-9, Bax, and Cyt-c, increase the expression of Bcl-2, and alleviate mitochondrial dysfunction and the mitochondrial apoptosis pathway in chicken lymphocytes [137]. Consistently, supplementing RSV can significantly improve the reduction in mitochondrial membrane potential induced by ZEA, activate the SIRT1/FOXO3a signaling pathway, decrease the Bax/Bcl-2 ratio, and alleviate ZEA-induced mitochondrial dysfunction and cell apoptosis [29]. At the same time, RSV can also attenuate damage to mitochondria caused by external stimuli by modulating the electron affinity of the electron transport chain and the activity of F0F1-ATPase [138].

**Figure 6:**
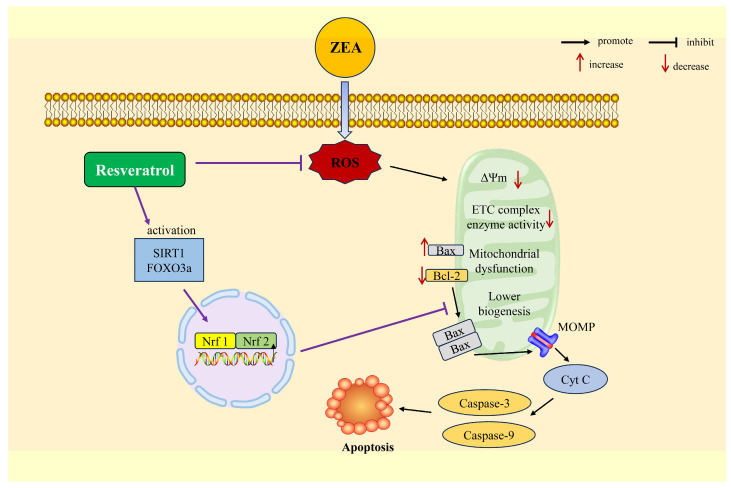
RSV attenuates ZEA-induced mitochondrial dysfunction and apoptosis by activating the SIRT1/FOXO3a signaling pathway and regulating mitochondrial function.

Figure 6 summarizes the molecular mechanisms of ZEA-induced mitochondrial dysfunction and apoptosis pathways, as well as the protective effect of RSV. ZEA can induce mitochondrial dysfunction and apoptosis through multiple molecular pathways, including the impact on mitochondrial membrane potential, mitochondrial membrane structural proteins, the electron transport chain, the TCA cycle, and oxidative phosphorylation, as well as the expression of proteins such as Bcl-2, Bax, Cyt C, Caspase-9, and Caspase-3. Furthermore, OS is a key factor in mitochondrial dysfunction [139]. Therefore, the inhibitory effect of RSV on ROS production may also play a key role. RSV can greatly improve the cytotoxicity and tissue damage caused by ZEA by targeting these sites. However, the specific molecular mechanisms of RSV on ZEA-induced mitochondrial dysfunction still need to be further explored in detail.

### 4.4. RSV Improves ZEA-Induced Autophagy and Mitochondrial Autophagy

Autophagy is a process of cellular self-phagocytosis, in which damaged proteins or organelles are enclosed in a double-membrane structure called autophagosomes, then transported to lysosomes for degradation, completing the recycling of cellular contents and maintaining and regulating cellular balance [140]. Research shows that dysfunction of autophagy can lead to organ inflammation, cancer, infectious diseases, immune dysfunction, and neurodegenerative diseases [141]. Autophagy can be activated under a variety of stress states such as hypoxia, nutrient deficiency, DNA damage, and OS, thus playing a protective role [142]. Previous studies have shown that exposure to ZEA can either promote or inhibit cellular autophagy, depending on the dosage or target organ of ZEA. Yang et al. found that feeding pigs with feed containing 0.15, 1.5, and 3.0 mg/kg of ZEA for 32 consecutive days significantly increased the expression levels of autophagy-related genes *LC3*, *Beclin1*, *ATG5*, *ATG7*, *ATG9*, and *p-AMPK*, as well as the anti-apoptotic gene *Bcl-2* in uterine tissues. The expression levels of *Bax* and *p-mTOR* were significantly decreased. Ultimately, autophagy was induced and apoptosis was inhibited by activating the AMPK/mTOR signaling pathway, leading to uterine hypertrophy [52].

Zhu et al. found that treatment of chicken granulosa cells with 20 μM ZEA for 24 h significantly increased the protein expression levels of LC3-II and Beclin1, while significantly reducing the protein level of P62. The expression levels of p-PI3K/PI3K, p-AKT/AKT, and p-mTOR/mTOR were significantly decreased. The MAPK family protein p-ERK1/2/ERK1/2 indicated that the PI3K/Akt/mTOR and ERK signaling pathways were involved in ZEA-induced autophagy. After treatment with autophagy inhibitors and activators CQ and RAP, it was found that autophagy could inhibit ZEA-induced apoptosis in chicken granulosa cells [54]. Liu et al. found that treatment of boar Sertoli cells with 25, 50, and 100 μM ZEA for 24 h resulted in elevated levels of ROS, inducing cell autophagy and significantly increasing the ratio of LC3-II/I, while significantly reducing the protein expression level of P62 [143]. However, researchers such as She found that giving male BALB/C mice an oral gavage dose of 40 mg/kg BW ZEA for 5 or 7 consecutive days significantly increased the expression levels of autophagy-related proteins Beclin1, ATG5, P62, and LC3 in testicular tissue, inducing cellular autophagy. However, the autophagic flux was blocked, leading to a decrease in sperm viability in male mice [144]. Previous studies have shown that RSV is a natural autophagy regulator [145]. RSV regulates cellular autophagy and autophagic flux by inhibiting the PI3K/Akt/mTOR signaling pathway [146], thus alleviating oxidative damage caused by various stress conditions [147]. The research shows that exposure to RSV significantly increases the protein expression levels of autophagy-related proteins LC3 and Beclin1. It induces autophagy and apoptosis through activation of the NGFR-AMPK-mTOR pathway. It was also found that protective autophagy is induced at concentrations lower than 55 μM [148]. Liu et al. found that RSV can decrease the protein expression levels of P62 and increase the protein expression levels of LC3-II/LC3-I. It inhibits autophagy of myocardial ischemia-reperfusion injury by regulating the MAPK/ERK mitogen-activated protein kinase kinase 1 (MEKK1)/JNK pathway, thus maintaining myocardial homeostasis [149].

**Figure 7:**
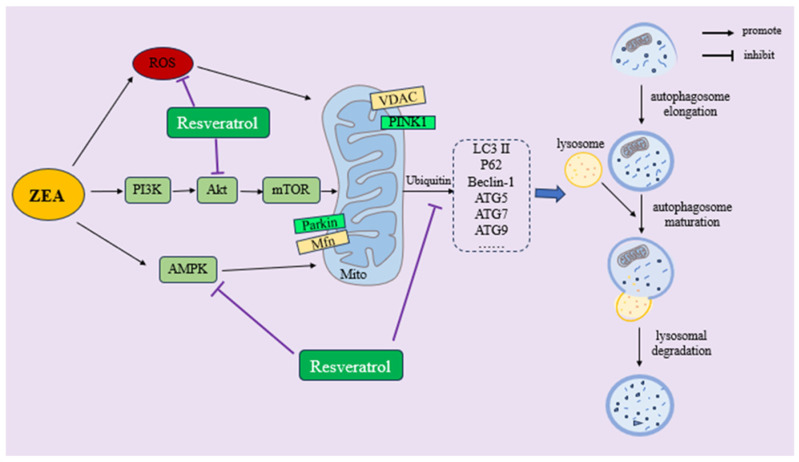
RSV regulates ZEA-induced mitochondrial autophagy and cellular autophagy through PI3K/Akt/mTOR and AMPK signaling pathways.

Mitophagy, which represents a type of selective autophagy, constitutes a unique manifestation of autophagy. It targets damaged or excessive mitochondria through the autophagic system and transports them to lysosomes for degradation, playing an important role in controlling and maintaining the quantity and quality of mitochondria [150]. Previous studies have shown that PINK1 and Parkin are involved in and maintain mitochondrial function through mitophagy [151]. PINK1 accumulates in mitochondria with reduced membrane potential. When mitochondrial function is disrupted, the expression levels of the intracellular PINK1 protein increase significantly. PINK1 accumulates and phosphorylates on the mitochondrial membrane, inducing the recruitment and activation of Parkin, thereby degrading damaged mitochondria [152]. Previous studies have found that exposure to 20μM ZEA significantly increases the protein expression levels of PINK1 and Parkin in porcine oocytes, enhances mitochondrial autophagy flux, disrupts mitochondrial ultrastructure, and increases ROS generation. After treatment with 2μM RSV, the protein expression levels of PINK1 and Parkin significantly decrease, the mitochondrial autophagy flux is alleviated, and ROS levels decrease [83]. Therefore, RSV can inhibit the generation of ROS to inhibit ZEA-induced cellular autophagy or mitophagy. Evidence suggests that supplementing RSV can provide protective effects by regulating cellular autophagy and mitophagy. Figure 7 shows the regulatory effect of RSV on ZEA-induced autophagy or mitophagy, but the detailed mechanism needs further investigation in experiments.

## 5. Conclusions

ZEA is one of the most contaminated mycotoxins and can cause neurotoxicity, immunotoxicity, hepatotoxicity, nephrotoxicity, reproductive toxicity, and cancer in humans and animals by inducing OS, inflammatory response, apoptosis, immunosuppression, etc. In recent years, scientists have been dedicated to finding natural, safe supplements to alleviate the toxicity caused by mycotoxins. It is worth noting that RSV mitigates the toxic effects caused by ZEA and has been shown in clinical trials to be safe and inexpensive. The molecular mechanisms underlying the protective effect of RSV on ZEA mainly involve signaling pathways such as Nrf2, NLRP3, TLR4, NF-κB, SIRT1/FOXO3a and PI3K/Akt/mTOR. It can inhibit the oxidative damage caused by ZEA and the inflammatory response, reduce the dysfunction of mitochondria, and scavenge the free radicals, as well as regulate the alteration of the microbial composition of the gut. However, RSV still faces challenges of low water solubility and rapid metabolism. Furthermore, research on the detoxification of ZEA by RSV has mainly been conducted in animal and in vitro cell experiments, but clinical evidence regarding its detoxifying effects on ZEA in humans is limited. Hence, further studies are essential to fully understand the detoxification capacity of RSV.

## Figures and Tables

**Table 1:** A summary of in vitro and animal models involving the protective effects of RSV on ZEA-induced toxicity.

Cell/Animals	Treatments	Regulated Effects of RSV	References
Human endometrial stromal cells	Cells were treated with 50 μM ZEA for 48 h and then with RSV for 24 h.	ZEA exhibited its inhibitory action through nuclear translocation of ERα. ZEA exposure led to dampened progress of decidualization. RSV administration restored impaired decidualization process by induction of antioxidative gene *glutathione peroxidase 3* (*GPX3*).	[80]
HEK293cells	HEK293 cells were first pretreated with 2 μM RSV for 24 h, and then the cells were treated with 80 μM ZEA for 24 h.	RSV alleviated ZEA-induced OS and apoptosis by recovering the activity of manganese superoxide dismutase (MnSOD), increased MMP and cell viability, decreased ROS, decreased the expression of pro-apoptotic genes, and activated the SIRT1/FOXO3a pathway.	[29]
Rat Sertoli cells	Sertoli cells were treated with 0, 5, 10, 20 μM ZEA and 5 μM RSV for 24 h.	The ZEA-induced cytotoxicity and decline in lactic acid production in SCs were alleviated by the use of RSV.	[31]
Porcine Oocyte	Porcine oocytes were exposed to 20 μM ZEA with or without 2 mM RSV.	RSV redressed ZEA-induced mitochondrial depolarization, OS, and apoptosis, and accelerated mitochondrial DNA copy during maturation, which improved embryonic development.	[83]
TM4 cells	TM4 cells were treated with 5 μM RSV and 20 μM ZEA for 24 h.	RSV pretreatment significantly reduced the expression of apoptosis-related proteins, increased cell viability, and reduced MDA and ROS levels by increasing Nrf2 nuclear translocation and HO-1 expression. RSV protected TM4 cells from ZEA-induced OS and apoptosis via PI3K/Akt-mediated activation of the Nrf2/HO-1 signaling pathway.	[82]
5-week-old male BALB/c mice	Mice were orally administrated with RSV at 50,100, or 200 mg/kg BW with ZEA at 40 mg/kg BW for 12 days. After treatment, jejunum tissue and serum were collected for assessment.	RSV pretreatment significantly reduced serum DAO, and D-lactate levels altered intestinal morphology and markedly restored TJ protein levels, intestinal goblet cell number, and *MUC-2* gene expression after ZEA challenge. RSV supplementation attenuates intestinal OS, inflammation, and intestinal mucosal barrier damage induced by ZEA exposure by modulating NF-κB and Nrf2/HO-1 signaling pathways.	[30]
Male Wistar rats (BW is in the range of 100–150 g)	Rats were injected intraperitoneally with 2 mg/kg ZEA once a week and gavaged with 5 mg/kg RSV daily for 3 weeks. Serum and liver and kidney tissues were collected.	RSV improves ZEA-induced alterations in serum biochemical markers (HGB, PLT, MCV, MPV, RSV), markers of OS (MDA, SOD), immunotoxicity (IgG, IgM), and biomarkers of DNA damage, prevents DNA damage, and regulates DNA repair.	[81]

## Data Availability

Not applicable.
